# Idiopathic mesenteric phlebosclerosis: A rare cause of chronic diarrhea

**DOI:** 10.1002/jgh3.12335

**Published:** 2020-04-11

**Authors:** Shun Fung Sze, Polly Woon Yee Lam, Jodis Ting Wa Lam, Cliff Chi Chiu Chung, Yuk Tong Lee

**Affiliations:** ^1^ Department of Medicine Queen Elizabeth Hospital Kowloon Hong Kong; ^2^ Department of Pathology Caritas Medical Centre Kowloon Hong Kong; ^3^ Department of Surgery The Chinese University of Hong Kong Hong Kong; ^4^ Department of Medicine and Therapeutics The Chinese University of Hong Kong Hong Kong

**Keywords:** Chinese herbal medicine, chronic diarrhea, idiopathic mesenteric phlebosclerosis

## Abstract

We report a 45‐year‐old healthy Chinese woman who presented with chronic diarrhea and iron deficiency anemia, with colonoscopy showing multiple ulcers from cecum to sigmoid on a background of dark‐purple mucosa. She was initially suspected to be suffering from inflammatory bowel disease, but the peculiar colonic biopsy findings and computed tomography (CT) imaging features, together with her habit of using Chinese herbal supplements, supported the rare diagnosis of idiopathic mesenteric phlebosclerosis.

## Graphical abstract

A 45‐year‐old healthy Chinese woman presented with chronic diarrhea and iron deficiency anemia, with colonoscopy showing multiple colonic ulcers on a background of dark‐purple mucosa. Biopsies showed a peculiar pattern of fibrosis, while CT abdomen showed bowel wall thickening and vascular calcifications along mesentery of right‐sided colon, leading to a diagnosis of idiopathic mesenteric phlebosclerosis.

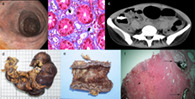

## Introduction

We report the clinical course of a 45‐year‐old healthy Chinese woman who presented with chronic diarrhea with occasional mucus and blood in stool. There was no evidence of gastrointestinal infection. Colonoscopy showed tiny ulcers and a background of dark‐purple mucosa. Colon biopsies and CT abdomen did not show typical features of inflammatory bowel disease. She regularly consumed Chinese herbs as a health supplement. The patient's history, histological, and radiological findings led to a diagnosis of idiopathic mesenteric phlebosclerosis (IMP), which was confirmed by gross pathology after operation.

## Case report

**Figure 1:**
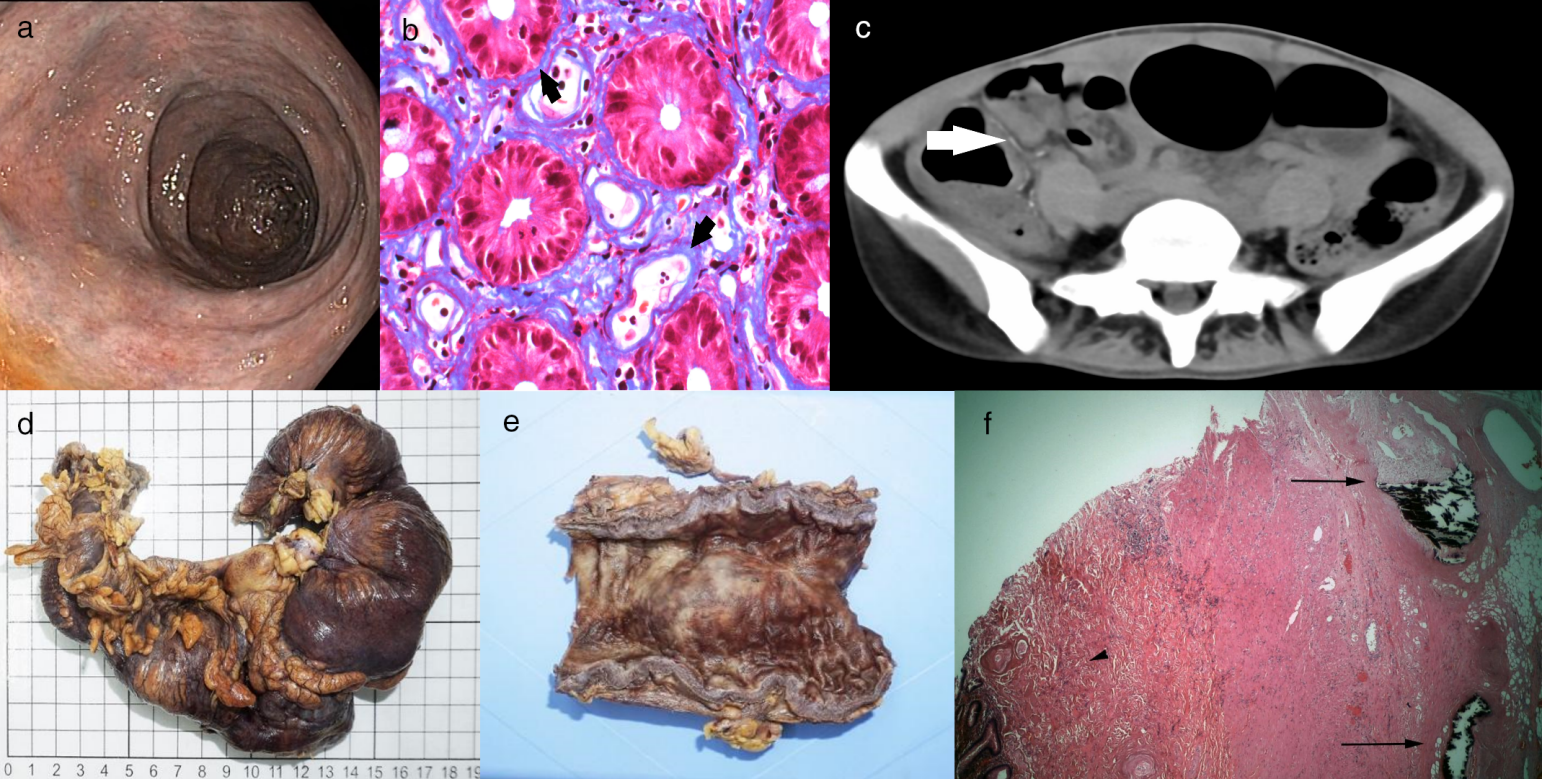
(a) Colonoscopy view showing the dark‐purple or cyanotic mucosa; (b) chromotrope aniline blue stain showed collagen deposition around crypts and especially around blood vessels; (c) CT scan showed curvilinear vascular calcifications over bowel wall and adjacent mesentery; (d) resected colon showed focal dilatation with its wall being dark purple in color; (e) cut section of colon showed thickened colonic wall with focal white fibrotic areas; and (f) microscopic section of the colon showed sclerosis and calcification of mesenteric veins (arrows) and fibrosis of mucosa and submucosa (arrow head).

A 45‐year‐old healthy Chinese woman presented with chronic diarrhea up to six times per day with occasional mucus and blood in stool. There was no abdominal pain. She consumed Chinese herbs as a health supplement for more than 10 years. She was afebrile, with normal blood pressure and pulse. Abdominal examination was unremarkable. Laboratory tests showed iron deficiency anemia with hemoglobin 9.1 g/dL, normal white cell count, and normal C‐reactive protein level. Stool culture was negative. Colonoscopy found tiny ulcers from cecum to sigmoid on a background of cyanotic mucosa (Fig. 1a). Colonic biopsies demonstrated ulcers, moderate inflammation, and a peculiar pattern of fibrosis. Chromotrope Aniline blue stain showed collagen deposition, especially around blood vessels (Fig. 1b). There was no evidence of amyloidosis, and Congo red stain was negative. CT abdomen showed diffuse bowel wall thickening from the cecum to sigmoid colon, and curvilinear calcifications over the bowel wall of cecum, ascending colon, and adjacent mesentery were suggestive of vascular calcifications (Fig. 1c), strongly supporting the diagnosis of IMP. 5‐aminosalicylic acid (ASA) and steroids were prescribed after her colonoscopy, and she stopped consuming Chinese herbs, resulting in symptomatic improvement. However, she developed intestinal obstruction and presented with repeated vomiting 3 years later, and right hemicolectomy was performed. The resected colon showed focal dilatation, with its wall being dark purple in color (Fig. 1d), and its cut section showed a thickened colonic wall with focal white fibrotic areas (Fig. 1e). Histology showed sclerosis and calcification of mesenteric veins and fibrosis of mucosa and submucosa (Fig. 1f), compatible with the diagnosis of IMP.

## Discussion

This patient suffered from chronic diarrhea and anemia, with colonoscopy showing multiple colonic ulcers, of which inflammatory bowel disease was initially suspected. It was not until after her CT was performed that phlebosclerotic colitis or IMP was suggested. Previous case reports on IMP^1^, ^2^ described that, in colonoscopy, the colonic mucosa would show a cyanotic or dark‐purple color with ulcerations and erosions. Colonic biopsies were indicative but not specific to IMP as the characteristic features, such as calcification and mural thickening of venous walls, were found in mesenteric veins. The distinctive feature was a CT scan showing thread‐like calcifications along the large bowel wall and mesenteric veins,^3^ commonly involving the right hemicolon. There is no established treatment for IMP. Previous reports found its association with chronic use of herbal medicine (e.g. geniposide) or Chinese medical liquor (e.g. Acanthopanax gracilistylus wine, which may contain geniposide, Angelica sinensis, and Cortex acanthopancis).^2^ Our patient reported long‐term consumption of Angelica sinensis, and she was advised to stop using all herbs. Her diarrhea improved with this conservative strategy. However, the preexisting venous occlusion that caused bowel wall thickening and sclerosis precipitated to ileus and intestinal obstruction. After right hemicolectomy, she then remained well and asymptomatic. Diagnosis of this rare condition requires a high index of suspicion together with good history taking and characteristic CT findings.
